# Coping with oil spills: oil exposure and anxiety among residents of Gulf Coast states after the Deepwater Horizon Oil Spill

**DOI:** 10.14324/111.444/ucloe.000035

**Published:** 2022-05-27

**Authors:** Zachary E. Goldman, John A. Kaufman, J. Danielle Sharpe, Amy F. Wolkin, Matthew O. Gribble

**Affiliations:** 1 Department of Environmental Health, Emory University Rollins School of Public Health, Atlanta, GA 30322, USA; 2 Department of Epidemiology, Emory University Rollins School of Public Health, Atlanta, GA 30322, USA; 3 National Center for Injury Prevention and Control, Centers for Disease Control and Prevention, Atlanta, GA 30345, USA; 4 Department of Epidemiology, University of Alabama at Birmingham School of Public Health, Birmingham, AL 35294, USA

**Keywords:** generalised anxiety, disaster recovery, mental health, emergency response, Gulf States Population Survey (GSPS)

## Abstract

In April 2010, a fatal explosion on the Deepwater Horizon drilling rig in the Gulf of Mexico resulted in the largest marine oil spill in history. This research describes the association of oil exposure with anxiety after the Deepwater Horizon Oil Spill and evaluates effect modification by self-mastery, emotional support and cleanup participation. To assess the impacts of the Deepwater Horizon Oil Spill, the Centers for Disease Control and Prevention (CDC) conducted the Gulf States Population Survey (GSPS), a random-digit-dial telephone cross-sectional survey completed between December 2010 and December 2011 with 38,361 responses in four different Gulf Coast states: Louisiana, Florida, Alabama and Mississippi. Anxiety severity was measured using the Generalised Anxiety Disorder (GAD) symptom inventory. We used Tobit regression to model underlying anxiety as a function of oil exposure and hypothesised effect modifiers, adjusting for socio-demographics. Latent anxiety was higher among those with direct contact with oil than among those who did not have direct contact with oil in confounder-adjusted models [β = 2.84, 95% confidence interval (CI): 0.78, 4.91]. Among individuals with direct contact with oil, there was no significant interaction between participating in cleanup activities and emotional support for anxiety (*p* = 0.20). However, among those with direct contact with oil, in confounder-adjusted models, participation in oil spill cleanup activities was associated with lower latent anxiety (β = −3.55, 95% CI: −6.15, −0.95). Oil contact was associated with greater anxiety, but this association appeared to be mitigated by cleanup participation.

## Introduction

In April 2010, the explosion of the Deepwater Horizon drilling rig in the Gulf of Mexico resulted in massive ecological and community harms leading to a $4 billion plea agreement with BP Exploration & Production, a $400 million criminal plea agreement with Transocean, a $14.9 billion civil settlement with BP Exploration & Production and a $159 million civil penalty ruling against Anadarko Petroleum Corporation [1]. Two thousand kilometres of coastline were impacted by the pollution from the spill [2], which was a complex mixture that often included oil as well as dispersants (chemicals that contain surfactants and/or solvent compounds) used in cleanup efforts [3,4]. There has been extensive research on the environmental impacts of the spill [5–7], as well as several public health impact assessments of nearby communities [8–11]. It was anticipated by public health authorities that there was potential for adverse mental health impacts on the affected community based on the experience of previous oil spills (e.g., smaller spills such as the *Hebei Spirit* tanker disaster in Korea [12] and *Exxon Valdez* in Alaska [13]), so to collect data on experiences pertaining to the oil spill and the mental health of the community in the wake of the Deepwater Horizon Oil Spill, the Substance Abuse and Mental Health Services Administration (SAMHSA) and the Centers for Disease Control and Prevention (CDC) developed the Gulf States Population Survey (GSPS) as a telephone survey administered to more than 38,000 adults of age ≥18 years old in affected Gulf Coast states (Louisiana, Mississippi, Alabama and Florida) from December 2010 to December 2011 [14].

The Deepwater Horizon Oil Spill had a large number of people involved in cleanup efforts [15–17]. There have been several investigations specifically focused on the mental health of participants in oil spill cleanup efforts following the Deepwater Horizon Spill, in particular evidence from the National Institute for Environmental Health Science’s Gulf Long-Term Follow-Up Study (GuLF) [16,18,19] that found complex patterns of association that speak to multiple mediating pathways that might act in different directions on health outcomes and sometimes lead to net harms of cleanup participation [20,21]. However, a previous analysis of GSPS data focused on depression outcomes of cleanup participation among persons exposed to oil and found a potentially ameliorative association [22]. Several studies have focused on the associations between mental health and oil spill cleanup participation by comparing cleanup participants to populations not involved with the disaster [18,23,24]. Few studies have compared cleanup participants to non-cleanup participants among populations impacted by the disaster. A recent study on the associations of oil contact and depression found that respondents with oil contact had lower depression severity if they participated in cleanup activities, compared to exposed individuals who did not participate in cleanup activities [22].

The primary objective of this cross-sectional study was to evaluate whether oil exposure was associated with the severity of anxiety after the Deepwater Horizon Oil Spill in the GSPS, and assess effect modification of that association by self-mastery (coping ability), emotional support and cleanup participation. A secondary objective was to contribute to the evidence base regarding oil spill cleanup participation impacts on mental health among persons exposed to oil.

## Methods

### Study population

Following the Deepwater Horizon Oil Spill, the Substance Abuse and Mental Health Services Administration (SAMHSA) and the Centers for Disease Control and Prevention (CDC) published a report titled, *Behavioral Health in the Gulf Coast Region Following the Deepwater Horizon Oil Spill*, which described the behavioural health of residents impacted by the Deepwater Horizon Oil Spill [25]. Included in the report was the GSPS, a random-digit-dial telephone survey conducted between December 2010 and December 2011 to assess the impacts of the Deepwater Horizon Oil Spill [26]. The survey was created to provide information on the mental health status of the coastal population in areas affected by the oil spill [14]. The GSPS, a publicly available, coded dataset, included 38,361 responses from individuals aged ≥18 years old residing in Louisiana, Florida, Alabama and Mississippi [14]. The majority of the interviews were conducted in 25 coastal counties or parishes within 32 miles (approximately 50 km) of fishing locations closed due to the oil spill [14]. To compare the results from coastal counties to non-coastal counties, 27,947 interviews were conducted in coastal counties and 10,414 interviews were conducted in non-coastal counties [14].

### Data description

There were 16 questions found in the GSPS that measured contact with oil from the Deepwater Horizon Oil Spill. Direct oil contact was assessed by the question, ‘Did you have direct contact with the oil from the Gulf oil spill? (yes/no)’. Questions from the 7-item Generalised Anxiety Disorder (GAD-7) questionnaire were also used to self-screen for moderate-to-severe cases of generalised anxiety [14]. Of the individuals contacted for the GSPS, 44.2% completed the interview [27]. The data for the GSPS were weighted to adjust for United States Census population estimates, and the survey design, sampling methods and weights were based on methods used for the Behavioral Risk Factor Surveillance System [25]. Analysis of this resource does not qualify as Human Subjects Research under 45 CFR 46.102 because this is a publicly available deidentified dataset.

### Analysis

**Figure 1:**
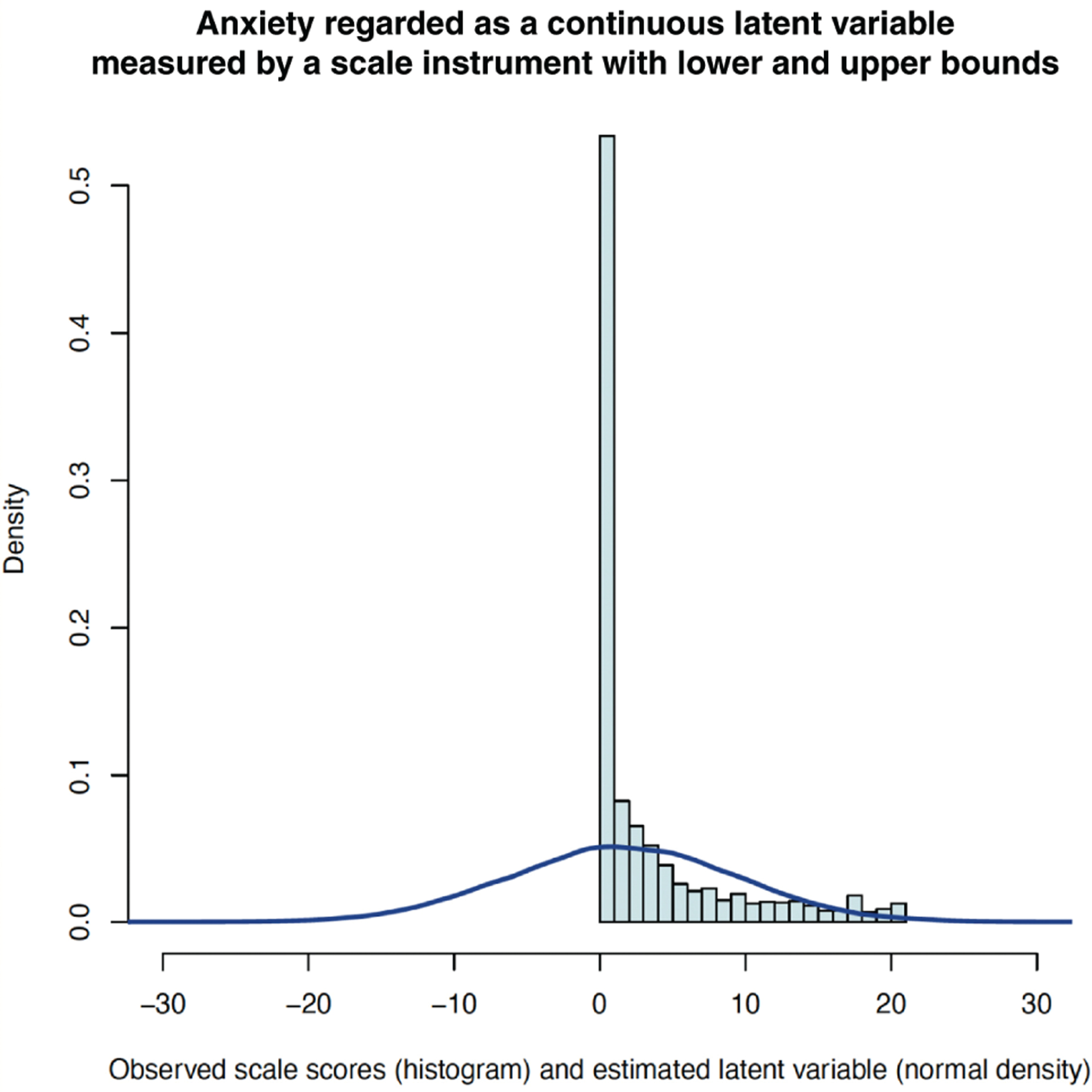
Latent anxiety and observed anxiety symptom scores in the GSPS.

In the GSPS, the observed GAD-7 scores of survey respondents could range from 0 to 21 for anxiety symptom severity [14]. We conceptualised mental health as being on a continuous gradient and therefore assumed there to be left-censoring of ‘negative’ anxiety (e.g., calmness, confidence) and right-censoring of extreme anxiety by the GAD-7 psychometric instrument. Tobit regression is a tool for modelling a latent dependent variable assumed to be normally distributed whose true values are censored by the measurement instrument [28]. Therefore, under the assumption that latent anxiety in the population was normally distributed rather than bounded between 0 and 21, Tobit regression was used to model underlying latent anxiety as a normally distributed latent variable. Figure 1 presents a histogram of observed GAD-7 scores along with modelled Tobit latent anxiety severity.

We adjusted models for age (continuous), gender (male/female), race (White/Black/other), Hispanic ethnicity (yes/no), smoking status (never/former/current), binge drinking (yes if over previous 30 days had consumed ≥ 5 drinks on one occasion if male or ≥ 4 drinks on one occasion if female), participating in any form of exercise (yes/no), marital status (married/not currently married) and employment status [employed (employed for wages, self-employed)/not employed (out of work, unable to work)/other (homemaker, student, retired)]. We further added interaction terms with how often people felt they had the emotional support they needed (always/usually/sometimes/rarely or never) and perceived self-mastery, a confirmatory factor analysis score derived from five items (strength of agreement with ‘I have little control over the things that happen to me’, ‘What happens to me in the future mostly depends on me’, ‘I can do just about anything I really set my mind to do’, ‘I am confident in my ability to handle unexpected problems’ and ‘When I need suggestions about how to deal with a personal problem, I know there is someone I can turn to’) [29].

In fully adjusted models, we tested for an interaction between direct contact with oil and participation in oil spill cleanup efforts. We also examined the association of cleanup participation with anxiety, controlling for the same confounders among the subpopulation with direct oil exposure, and tested for interactions with emotional support and self-mastery.

Survey estimation methods were used to account for the survey design with singleton primary sampling units treated as certainty units [30,31]. Missing data were handled by multiple imputation by chained equations with 80 imputations using all modelled variables (GAD-7 score, race, age, Hispanic ethnicity, direct oil contact, marital status, employment status, exercise, binge drinking, self-mastery, oil cleanup participation, smoking status and level of emotional support) [32]. All analyses were conducted using Stata/IC 16.1 (StataCorp, College Station, TX, USA).

## Results

### Population and sample demographics

Characteristics of the population are provided in Table 1, according to whether they had direct contact with oil from the Deepwater Horizon Oil Spill and whether they participated in cleanup activities. These estimates incorporate multiple imputation and survey estimation for population inference. Characteristics of the sample population according to direct oil contact and participation in cleanup activities during the Deepwater Horizon Oil Spill are provided in Table 2.

### Oil contact with anxiety

In fully adjusted models, latent anxiety was higher (β = 2.84, 95% confidence interval (CI): 0.78, 4.91, *t*-test *p* value = 0.01) among individuals with directly contact with oil compared to individuals not with direct contact to oil (Table 3). There was no heterogeneity in the oil-anxiety association across levels of emotional support (always, usually, sometimes and rarely or never) (3-degrees-of-freedom F test: *p* value = 0.38), with the lowest β-coefficient of 0.96 (95% CI: −0.84, 2.76) for ‘usually supported’ and the highest β-coefficient of 4.72 (95% CI: 0.53, 8.92) for ‘always supported’ (Table 4). There was also no significant modification of the association between oil exposure and anxiety by self-mastery (*t*-test *p* value = 0.12). Among those with direct contact to oil, individuals with lower self-mastery scores (minimum in sample: −4.04) had higher latent anxiety (β = 9.15, 95% CI: 0.03, 18.28) compared to individuals with higher self-mastery scores (maximum in sample: 3.31) (β = −3.13, 95% CI: −9.72, 3.45).

### Cleanup participation and anxiety among those with oil contact

There was, however, a significant antagonistic interaction between direct oil exposure and participation in cleanup activities for latent anxiety (*t*-test *p* value < 0.01). Among individuals with direct contact with oil in fully adjusted models, cleanup participation was associated with lower latent anxiety (β = −3.55, 95% CI: −6.15, −0.95) (Table 5). Among individuals with direct contact with oil, there was no significant interaction between participating in cleanup activities and levels of emotional support (always, usually, sometimes, rarely or never) (3-degrees-of-freedom F test: *p* value = 0.20), with the lowest β-coefficient of −4.08 (−8.53, 0.37) for ‘usually’ and the highest β-coefficient of 1.55 (−2.59, 5.68) for ‘sometimes’ (Table 6).

Among those with direct contact to oil and who participated in cleanup activities, individuals with lower self-mastery scores (minimum in sample: −4.04) had less latent anxiety (β = −12.87, 95% CI: −24.98, −0.76) compared to individuals with higher self-mastery scores (maximum in sample: 3.31) (β = 4.69, 95% CI: −5.66, 15.05). Thus, although the interaction term between cleanup participation with self-mastery among those with direct contact with oil was not statistically significant (*t*-test *p* value = 0.12), there is suggestive evidence that participation may have had a larger benefit among individuals with lower self-mastery.

## Discussion

### Oil contact and increased anxiety

This study found positive associations between oil contact and anxiety among residents of Gulf States after the Deepwater Horizon Oil Spill. These results are consistent with previous findings from studies examining associations between oil exposure and acute mental health impacts [11,18,22,33,34]. The implications for longer-term mental health are unclear; one recent study found that physical exposure to the Deepwater Horizon Oil Spill was not associated with measures of psychological resiliency for several years among adults with children [35], while another study found that later mental health in the community might be a function of cumulative exposure to other disasters as well (e.g., Hurricane Katrina) not only the Deepwater Horizon Oil Spill [36]. Our findings that exposed individuals with lower self-mastery scores had greater mental health impacts also agree with studies demonstrating the positive associations between high levels of self-mastery and improved mental health [37].

### Oil contact, cleanup participation and anxiety

We found that the association between oil exposure and latent anxiety is diminished among participants in oil cleanup activities. To the best of our knowledge, this is the first study to examine associations between anxiety and participation in oil spill cleanup activities within populations directly affected by the oil spill. These findings agree with a recent study examining the association between depression and participating in oil spill cleanup activities compared to not participating in these activities within populations directly affected by the oil spill [22]. This previous study also found associations between oil contact and mental health effects that were attenuated among the persons who participated in oil spill cleanup efforts [22].

### Volunteering, cleanup participation and mental health; similar findings in literature

Although this paper is the first to examine the relationship between oil exposure and anxiety by cleanup participation, prior studies have shown associations between general volunteering and mental health. In a longitudinal study conducted in the United Kingdom, individuals who engaged in volunteering regularly appeared to have higher levels of mental well-being compared to those who never volunteered [38]. A 2017 cross-sectional study found that combat veterans who volunteered in disaster relief social service organisations reported positive mental health responses as a result of helping those in need [39]. A previous study examining public servants working in the area affected by the Great East Japan Earthquake of 2011 also reported that engaging in disaster-related work may reduce mental health distress among workers [40].

### Study strengths and limitations

The main strengths of this paper were the use of a large, representative sample and the ability to compare different populations affected by the oil spill. However, because this was a cross-sectional survey, there was potential for reverse causality between cleanup participation and anxiety symptoms, both assessed after the oil spill [20]. It is also unknown whether respondents who participated in oil spill cleanup activities were healthier at baseline and had fewer mental health conditions. As the GSPS was a random-digit-dial telephone survey, the associations may introduce bias due to potential effects of recall bias or social desirability bias among the study participants [41].

Although the associations between cleanup participation and anxiety were adjusted for several factors related to anxiety and socio-demographics, confounding by unmeasured variables is possible. A recent study examining determinants of participating in oil spill cleanup activities found positive associations between cleanup participation and proximity to affected areas [15]. Approximately 4.7% of residents in affected Gulf communities participated in the oil spill cleanup activities, and most were young men in excellent physical health [15]. These individuals residing in affected communities may have been easily mobilised and motivated to help their affected communities by participating in cleanup activities [15].

## Implications and conclusions

It is possible that volunteering in disaster relief efforts could serve as an ameliorative intervention for mental health impacts after disasters occur. By responding to major disasters and working as a community, a sense of belonging or contribution may create a protective response for mental health [40]. Additionally, it has been shown that individuals who experience a disaster may benefit from the resources of their social networks [42]. We think it is plausible that participation in disaster-related response activities may give people a feeling of partial control over the consequences of an event that was otherwise out of their control. Increasing participation in disaster-related cleanup activities could also increase resilience and recovery of individuals and the surrounding community [15].

Although this cross-sectional study is limited in its ability to draw firm causal inferences, the findings from this paper raise the possibility that encouraging affected individuals to volunteer during oil spill disasters may mitigate the harmful effects of oil exposure. Further research, such as prospective evidence on the impacts of post-oil exposure cleanup participation on mental health outcomes, is needed.

## Figures and Tables

**Table 1.** Estimated characteristics (point estimates and 95% CI) of the population according to direct oil contact and participation in cleanup activities during the Deepwater Horizon oil spill. These estimates incorporate MI and survey estimation for population inference

Characteristic	Direct oil contact	No direct oil contact	Participated in cleanup activities	Did not participate in cleanup activities
Gender (%)				
Female	46 (38, 53)	52 (50, 54)	44 (33, 54)	52 (50, 54)
Male	54 (47, 62)	48 (46, 50)	56 (46, 67)	48 (46, 50)
Mean age (years)	43 (40, 46)	49 (48, 49)	39 (35, 42)	49 (48, 50)
Mean GAD-7 score	6 (5, 7)	4 (3, 4)	3 (3, 4)	4 (3, 4)
Race (%)				
White	76 (67, 85)	69 (67, 72)	72 (61, 83)	70 (67, 72)
Black	11 (7, 14)	20 (19, 22)	18 (8, 28)	20 (18, 22)
Other	13 (4, 22)	10 (8, 12)	10 (4, 17)	10 (9, 12)
Hispanic ethnicity (%)				
Yes	13 (4, 22)	11 (9, 13)	8 (1, 15)	12 (10, 13)
No	87 (78, 96)	89 (87, 91)	92 (85, 99)	88 (87, 90)
Employment (%)				
Employed	60 (52, 68)	52 (50, 54)	64 (53, 75)	52 (50, 54)
Not employed	16 (10, 21)	15 (14, 17)	12 (6, 18)	16 (14, 17)
Other	24 (18, 31)	32 (30, 35)	24 (13, 35)	32 (30, 34)
Marital status (%)				
Married	51 (43, 59)	52 (50, 54)	43 (32, 53)	52 (50, 55)
Not married	49 (41, 57)	48 (46, 50)	57 (47, 68)	48 (45, 50)
Exercise (%)				
Yes	82 (76, 88)	73 (71, 75)	89 (83, 95)	73 (71, 75)
No	18 (12, 24)	27 (25, 29)	11 (5, 17)	27 (25, 29)
Binge drinking (%)				
Yes	23 (17, 28)	15 (13, 16)	29 (19, 38)	15 (13, 16)
No	77 (72, 83)	85 (84, 87)	71 (62, 81)	85 (84, 87)
Smoking (%)				
Never	52 (44, 60)	55 (52, 57)	50 (39, 60)	55 (52, 57)
Former	21 (15, 27)	24 (22, 26)	22 (13, 32)	24 (22, 25)
Current	27 (20, 33)	22 (20, 24)	28 (19, 37)	22 (20, 24)
Emotional support (%)				
Always	40 (32, 48)	50 (48, 52)	53 (42, 63)	49 (47, 51)
Usually	33 (26, 40)	27 (25, 29)	32 (23, 41)	27 (25, 29)
Sometimes	16 (11, 21)	14 (13, 16)	6 (2, 10)	15 (13, 16)
Rarely or never	11 (6, 16)	9 (7, 10)	9 (3, 14)	9 (7, 10)

Abbreviations: CI: confidence intervals; MI, multiple imputation.

**Table 2.** Characteristics of the sample stratified by direct oil contact and participation in cleanup activities during the Deepwater Horizon oil spill. The numbers in rows that show (%) are N and percent, the numbers without (%) are mean and SD

Characteristic	Direct oil contact	No direct oil contact	Participated in cleanup activities	Did not participate in cleanup activities
Gender (%)				
Female	1,985 (55%)	21,300 (63%)	740 (47%)	22,698 (63%)
Male	1,640 (45%)	12,578 (37%)	835 (53%)	13,459 (37%)
Mean age (years)	3,625 (50)	33,878 (56)	1,575 (47)	36,157 (56)
Mean GAD-7 score	3,539 (5)	33,108 (3)	1,548 (4)	35,318 (3)
Race (%)				
White	2,983 (82%)	26,160 (77%)	1,289 (82%)	28,005 (78%)
Black	428 (12%)	5,910 (17%)	172 (11%)	6,234 (17%)
Other	210 (6%)	1,751 (5%)	112 (7%)	1,860 (5%)
Hispanic ethnicity (%)				
Yes	3,496 (97%)	32,749 (97%)	1,518 (97%)	34,948 (97%)
No	111 (3%)	962 (3%)	52 (3%)	1,024 (3%)
Employment (%)				
Employed	2,063 (57%)	15,510 (46%)	1,033 (66%)	16,654 (46%)
Not employed	615 (17%)	4,423 (13%)	215 (14%)	4,879 (14%)
Other	938 (26%)	13,856 (41%)	323 (21%)	14,531 (40%)
Marital status (%)				
Married	2,114 (59%)	17,709 (52%)	882 (56%)	19,045 (53%)
Not married	1,496 (41%)	16,027 (48%)	683 (44%)	16,963 (47%)
Exercise (%)				
Yes	2,868 (80%)	24,251 (72%)	1,299 (83%)	25,992 (72%)
No	732 (20%)	9,400 (28%)	265 (17%)	9,925 (28%)
Binge drinking (%)				
Yes	2,857 (80%)	29,550 (88%)	1,229 (80%)	31,366 (88%)
No	706 (20%)	3,889 (12%)	314 (20%)	4,322 (12%)
Smoking (%)				
Never	1,754 (49%)	17,927 (54%)	777 (50%)	19,018 (53%)
Former	939 (26%)	9,003 (27%)	368 (24%)	9,627 (27%)
Current	882 (25%)	6,513 (19%)	405 (26%)	7,053 (20%)
Emotional support (%)				
Always	1,570 (44%)	16,622 (50%)	743 (48%)	17,556 (49%)
Usually	1,114 (31%)	9,526 (28%)	494 (32%)	10,203 (29%)
Sometimes	543 (15%)	4,864 (15%)	208 (13%)	5,237 (15%)
Rarely or never	35 (10%)	2,490 (7%)	119 (8%)	2,760 (8%)

**Table 3.** Differences in anxiety (β) with direct contact with oil in communities participating in the GSPS

Model	β (95% CI)
Model 1: Unadjusted association	3.51 (1.79, 5.24)
Model 2: Adjusted for age, race, Hispanic ethnicity, gender	3.35 (1.60, 5.09)
Model 3: Further adjusted for exercise, smoking, and binge drinking	3.42 (1.61, 5.24)
Model 4: Further adjusted for employment and marital status	3.32 (1.50, 5.16)
Model 5: Further adjusted for self-mastery and emotional support	2.84 (0.78, 4.91)

Abbreviations: CI, confidence interval; GSPS, Gulf States Population Survey.

**Table 4.** Differences in anxiety (β) with direct contact with oil, by frequency of emotional support, in communities participating in the GSPS

Emotional support	Proportion of population (95% CI)	β (95% CI)
Always	0.49 (0.47, 0.52)	4.72 (0.53, 8.92)
Usually	0.27 (0.26, 0.29)	0.96 (−0.84, 2.76)
Sometimes	0.15 (0.13, 0.16)	1.89 (−0.46, 4.23)
Rarely or never	0.09 (0.07, 0.10)	3.10 (−0.51, 6.71)

Abbreviations: CI, confidence interval; GSPS, Gulf States Population Survey.

**Table 5.** Differences in anxiety (β) between individuals participating in oil spill cleanup activities compared to individuals not participating in oil spill cleanup activities, among individuals with direct contact with oil, in communities participating in the GSPS

Model	β (95% CI) among persons with direct oil contact	β (95% CI) among persons without direct oil contact
Model 1: Unadjusted association	−3.48 (−6.81, −0.15)	−0.35 (−2.30, 1.60)
Model 2: Adjusted for age, race, Hispanic ethnicity, gender	−3.18 (−6.16, −0.19)	−0.83 (−2.91, 1.24)
Model 3: Further adjusted for exercise, smoking, and binge drinking	−3.25 (−6.16, −0.33)	−0.56 (−2.49, 1.37)
Model 4: Further adjusted for employment and marital status	−3.47 (−6.12, −0.82)	−0.30 (−2.19, 1.60)
Model 5: Further adjusted for self-mastery and emotional support	−3.55 (−6.15, −0.95)	0.30 (−1.56, 2.17)

Abbreviations: CI, confidence interval; GSPS, Gulf States Population Survey.

**Table 6.** Associations between oil spill cleanup participation and anxiety, by frequency of emotional support, in communities participating in the GSPS

Emotional support	Proportion of population (95% CI)	β (95% CI)
Always	0.49 (0.47, 0.52)	−3.83 (−7.47, −0.19)
Usually	0.27 (0.26, 0.29)	−4.08 (−8.53, 0.37)
Sometimes	0.15 (0.13, 0.16)	1.55 (−2.59, 5.68)
Rarely or never	0.09 (0.07, 0.10)	−3.31 (−9.03, 2.40)

Abbreviations: CI, confidence interval; GSPS, Gulf States Population Survey.

## Data Availability

The datasets generated during and/or analysed during the current study are available from the corresponding author on reasonable request.
